# Electrodeposited Porous Mn_1.5_Co_1.5_O_4_/Ni Composite Electrodes for High-Voltage Asymmetric Supercapacitors

**DOI:** 10.3390/ma10040370

**Published:** 2017-03-31

**Authors:** Guan-Ting Pan, Siewhui Chong, Thomas C.-K. Yang, Chao-Ming Huang

**Affiliations:** 1 Department of Chemical Engineering and Biotechnology, National Taipei University of Technology, Taipei 106, Taiwan; t6679013@gmail.com (G.-T.P.); ckyang@ntut.edu.tw (T.C.-K.Y.); 2 Department of Chemical and Environmental Engineering, University of Nottingham, Selangor 43500, Malaysia; faye.chong@nottingham.edu.my; 3 Department of Materials Engineering, Kun Shan University, Tainan 710, Taiwan

**Keywords:** spinel, Ni foam, electrodeposition, gel electrolyte, asymmetric supercapacitor

## Abstract

Mesoporous Mn_1.5_Co_1.5_O_4_ (MCO) spinel films were prepared directly on a conductive nickel (Ni) foam substrate via electrodeposition and an annealing treatment as supercapacitor electrodes. The electrodeposition time markedly influenced the surface morphological, textural, and supercapacitive properties of MCO/Ni electrodes. The (MCO/Ni)-15 min electrode (electrodeposition time: 15 min) exhibited the highest capacitance among three electrodes (electrodeposition times of 7.5, 15, and 30 min, respectively). Further, an asymmetric supercapacitor that utilizes (MCO/Ni)-15 min as a positive electrode, a plasma-treated activated carbon (PAC)/Ni electrode as a negative electrode, and carboxymethyl cellulose-lithium nitrate (LiNO_3_) gel electrolyte (denoted as (PAC/Ni)//(MCO/Ni)-15 min) was fabricated. In a stable operation window of 2.0 V, the device exhibited an energy density of 27.6 Wh·kg^−1^ and a power density of 1.01 kW·kg^−1^ at 1 A·g^−1^. After 5000 cycles, the specific energy density retention and power density retention were 96% and 92%, respectively, demonstrating exceptional cycling stability. The good supercapacitive performance and excellent stability of the (PAC/Ni)//(MCO/Ni)-15 min device can be ascribed to the hierarchical structure and high surface area of the (MCO/Ni)-15 min electrode, which facilitate lithium ion intercalation and deintercalation at the electrode/electrolyte interface and mitigate volume change during long-term charge/discharge cycling.

## 1. Introduction

Global warming and particulate air pollution caused by fossil fuel combustion have become important issues. Fine particulate matter, under 2.5 μm in diameter (PM2.5), has been reported to impact the respiratory system [1]. Emissions of PM2.5 are partly due to energy production and consumption, and are associated with fossil fuel burning, industrial combustion processes, and vehicle emissions [2]. With concerns regarding PM2.5 levels, there is an urgent need for a clean and renewable energy source [3]. However, various renewable energy technologies, such as solar and wind, cannot produce a constant amount of energy. Thus, studies on renewable energy conversion and storage are imperative for the development of renewable energy technologies.

For energy conversion/storage systems, rechargeable Li-ion batteries have received a lot of attention. However, safety issues and a long charge time hinder their usefulness [4]. Compared to rechargeable Li-ion batteries, supercapacitors are much safer and have higher power density, much longer cycle lifetimes, and much shorter charging time, but much lower energy density [5]. To compete with Li-ion batteries, the most important issue for supercapacitor research is to achieve high energy density while maintaining high power density and high cyclability. Energy density can be expressed as *E* = 0.5*CV*^2^, where *C* is the specific capacitance and *V* is the operating voltage window. The energy density of a supercapacitor can, thus, be increased by increasing the specific capacitance or broadening the voltage window [4]. The most effective method is to combine a battery-type positive electrode and a capacitor-type negative electrode with a suitable electrolyte to create an asymmetric supercapacitor, which effectively increases both the energy and power capability of the supercapacitor in energy conversion and storage for renewable energy.

For the positive electrode, conducting polymers and pseudocapacitance materials based on transition-metal oxides/hydroxides are widely used. Conducing polymers have been used to create durable and flexible supercapacitors due to their low cost, light weight, flexibility, and excellent electrochemical properties [6]. Transition-metal oxides/hydroxides with two or three metal compounds, such as MnFe_2_O_4_ [7], Ni-Zn-Co oxide [8], and Ni-Co hydroxide [9], have been used as positive electrode materials due to their abundant structural defects and fast redox reactions. Among multi-metal compounds, spinel-type manganese-cobalt oxides exhibit superior electronic conductivity and electrochemical performance compared to those of either manganese oxides or cobalt oxides due to their variable valence states and structural flexibility during electrochemical redox reactions [10,11,12]. For example, the presence of Co in Mn_1.5_Co_1.5_O_4_ (MCO) tunes the Mn oxidation state, Mn^4+^/Mn^3+^, through an internal redox process, making MCO a promising electrode material for supercapacitors and batteries [13,14]. MCO is, thus, selected as the positive electrode in the present study.

For the negative electrode, carbon-based materials are widely applied for providing electrical double-layer capacitance (EDLC) to boost the power density of supercapacitors. Since EDLC arises from the electrostatic attraction between the electrolyte and the electrode surface, a large surface area and special pore shape and structure with a well-controlled pore size distribution are beneficial for EDLC. By using metal-organic frameworks (MOFs) as precursors, the controlled porous architectures of mesoporous MOF-derived carbons can be tailored to achieve sufficiently high energy density and power density for microelectronics and portable device applications [15]. In the present study, high-surface-area activated carbon (AC) pre-treated with air plasma to enhance its wetting ability is used as the negative electrode material.

Since energy density is proportional to the square of the cell voltage, it can be effectively increased by broadening the voltage window, which can be achieved by using an appropriate electrolyte. The requirements for such an electrolyte include a wide voltage window, high ionic concentration, and low cost [5]. Aqueous electrolytes, such as strong bases (KOH) and strong acids (H_2_SO_4_), are widely applied to supercapacitors because of their low cost, ease of use, high conductivity, and non-flammability. Since water decomposes at 1.23 V, the operating voltage of capacitors that use aqueous electrolytes cannot exceed 1.2 V. In addition, the water splitting reaction for hydrogen and oxygen evolution in acidic and alkaline electrolytes occurs readily during the high catalytic activity of metal oxide electrodes, resulting in poor cycling stability [16]. In contrast, an operating voltage of up to 4 V can be achieved with organic electrolytes. By combining small ether-substituted quaternary ammonium salts with tetrafluoroborate as organic electrolytes, EDLC can be remarkably enhanced [17]. Another promising electrolyte is a gel electrolyte, which is prepared by entrapping a liquid electrolyte within a polymeric network. Chodankar et al. [18] used polyvinyl alcohol polymer combined with a lithium perchlorate salt as a gel electrolyte. They reported that MnO_2_-based flexible solid-state supercapacitors with the gel electrolyte had an operating potential window of 1.2 V and an energy density of 15 Wh·kg^−1^ with extended cycling stability (up to 2500 cycles). Using a polymer gel electrolyte instead of a liquid electrolyte stops the dissolution of the active electrode material into the electrolyte, improving cycling stability [19].

In the present work, an asymmetric supercapacitor was assembled utilizing an MCO/Ni positive electrode, a plasma-treated activated carbon (PAC)/Ni negative electrode, and a carboxy methyl cellulose (CMC)-LiNO_3_ gel electrolyte. Porous MCO film was directly grown on the backbone of three-dimensional (3D) Ni foam without a polymer binder to reduce the contact resistance between the MCO and Ni foam via the electrodeposition method. To create a gel electrolyte, we adopted carboxy methyl cellulose (CMC), a kind of polysaccharide, to act as the host material, LiNO_3_ to provide Li^+^ for conduction, and H_2_O as the medium for ionic conduction. The effect of deposition time on the morphology, structure, and electrochemical properties of as-obtained MCO/Ni electrodes was explored. Tailoring the deposition time prior to assembly enabled optimization of the textural properties and electrochemical performance. (MCO/Ni)-15 min (deposition time: 15 min) had the highest specific capacitance among three samples (deposition time of 7.5, 15, and 30 min, respectively). Furthermore, an asymmetric supercapacitor with (PAC/Ni)//(MCO/Ni)-15 min had an operating potential window of 2.0 V and an energy density of 27.6 Wh·kg^−1^ at a power density of 1.01 kW·kg^−1^. After 5000 cycles the specific energy density retention and power density retention were 96% and 92%, respectively, indicating excellent cycling stability.

## 2. Experiments

### 2.1. Preparation of MCO/Ni Electrodes and PAC/Ni Electrodes

MCO films were deposited onto Ni foam via the potentiostatic electrodeposition technique. For pre-treatment, Ni foam was ultrasonically degreased in acetone for 30 min, etched using 2 M HCl for 20 min, washed with H_2_O, and finally dried in an oven at 110 °C for 3 h. The typical procedure of the co-electrodeposition of Mn and Co was as follows: Mn(CH_3_COOH)_2_·4H_2_O (0.006 mol) and Co(CH_3_COOH)_2_·4H_2_O (0.012 mol) were mixed with deionized water (250 mL) and stirred to make a solution. Then, Na_2_SO_4_ (0.01 mol) was added to the above homogeneous solution. Na_2_SO_4_ was used to prevent particle aggregation and to serve as a morphology-directing agent. All depositions were carried out at room temperature using an electrochemical workstation (CHI 660B, CH Instruments, Austin, TX, USA) with a three-electrode setup, which consisted of Ni foam, Pt foil, and a saturated calomel electrode (SCE) as the working, counter, and reference electrodes, respectively. The electrodeposition of Mn and Co mixed oxide onto Ni foam was carried out by applying +0.5 V (SCE) for 450, 900, and 1800 s, respectively. After deposition, the MCO/Ni electrodes were washed with deionized water, dried, and annealed at 400 °C for 2 h. These electrodes are denoted (MCO/Ni)-7.5 min, (MCO/Ni)-15 min, and (MCO/Ni)-30 min, respectively.

PAC/Ni electrodes were fabricated by first mixing PAC (75 wt %), with a Brunauer-Emmett-Teller (BET) surface area of 999 m^2^·g^−1^, KS-6 carbon (10 wt %), super-P carbon (10 wt %), and polyvinylidene fluoride (5 wt %), which was dispersed in N-methyl-2-pyrrolidinone (NMP) and 2-propanol. The prepared slurry was pressed on a piece of Ni foam under a pressure of 10.0 MPa for 10 s and dried under vacuum at 110 °C for 1 h.

### 2.2. Preparation of Gel Electrolyte and Asymmetric Supercapacitor Assembly

To prepare the CMC-based gel electrolyte, 2 g of CMC was dissolved in deionized water at a temperature of 75 °C under constant stirring. After complete dissolution of CMC, 2 g of LiNO_3_ was added into the prepared solution and stirred at room temperature until the formation of a viscous solution.

Asymmetric supercapacitors were fabricated using polypropylene as the separator, CMC-LiNO_3_ as the gel electrolyte, stainless steel as the collector, MCO/Ni as the positive electrode, (PAC)/Ni as the negative electrode, and laminated aluminum foil as the outer package. In the asymmetric supercapacitors, the mass loadings of MCO and PAC were 4 and 8 mg·cm^−^^2^, respectively.

### 2.3. Sample Characterization and Electrochemical Measurements

The characterization of the crystal structure via X-ray diffraction (XRD) patterns of the obtained samples was conducted using an X-ray diffractometer (PANalytical X’Pert PRO, PANalytical, Eindhoven, The Netherland) with Cu radiation (λ = 0.15418 nm). The morphology of MCO was observed using scanning electron microscopy (SEM, JEOL JSM-6700F, JEOL, Peabody, MA, USA). The textural properties of the MCO powders, scratched from the MCO/Ni composite, were determined using a volumetric sorption analyzer (Micromeritics ASAP 2020, Micromeritics Instrument Corporation, Norcross, GA, USA). The BET specific surface area and pore volume were determined from an N_2_ adsorption/desorption isotherm recorded at −196 °C and the pore size distribution was obtained using the Barrett-Joyner-Halenda (BJH) model. All measurements, including cyclic voltammetry (CV), galvanostatic charge/discharge (GCD), and electrochemical impedance spectroscopy (CHI 6273E, CH Instruments), were carried out on an electrochemical workstation (CHI 660B, CH Instruments) to assess the electrochemical properties of the electrodes for fabricating an asymmetric supercapacitor.

The specific capacitance *C* (F·g^−1^) of the MCO/Ni electrodes was calculated from the integral of the CV curves:

(1) $$C=\frac{1}{m\nu (V_{c}-V_{a})}\int_{V_{a}}^{V_{c}}I(V)dV$$

where *m* is the mass of MCO, *ν* is the potential scan rate (mV·s^−1^), *V*_c_ − *V*_a_ is the working voltage window, and *I*(*V*) is the response current (mA).

The long-term GCD performance of the asymmetric supercapacitor was evaluated using a battery testing station (580 Battery Test System, Scribner Associates Inc., Southern Pines, NC, USA) in the range of 0–2.0 V at 1 A·g^−1^. The specific capacitance *C* was calculated as *C* = [(*I* × Δ*t*)/(Δ*V* × *m*)], where *I* is the discharging current, Δ*t* is the discharging time, Δ*V* is the discharging voltage difference, and *m* is the mass of the loaded active materials. The EIS measurements were obtained by applying an AC voltage with a 5-mV amplitude in a frequency range of 0.01 Hz to 100 kHz at open-circuit potential.

To calculate the specific energy density (E) and specific power density (P) of the asymmetric supercapacitor, the following equations were used:

E = 0.5 CV^2^ (Wh·kg^−1^) (2)

P = E/t (W·kg^−1^) (3)

## 3. Results and Discussion

### 3.1. Characterization of MCO

#### 3.1.1. Structural Studies of MCO

**Figure 1:**
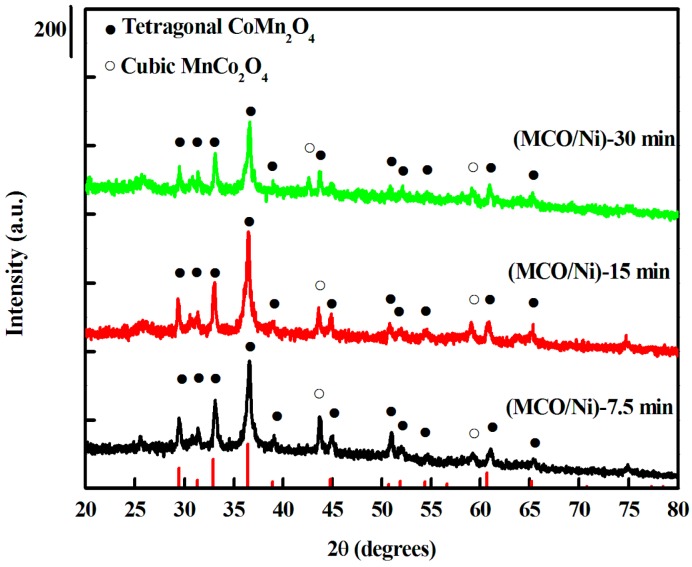
XRD patterns of Mn_1.5_Co_1.5_O_4_ powders scratched from MCO/Ni composite electrodes.

XRD patterns of manganese-cobalt oxide powders scratched from MCO/Ni electrodes were obtained. As depicted in Figure 1, the XRD patterns exhibit ten significant peaks, namely four strong peaks at 29.4°, 32.9°, 36.4 °, and 43.7°, and six weak peaks near 31.3°, 38.9°, 44.8°, 50.6°, 59.1°, and 60.7°, respectively. The peaks at 29.4°, 31.3°, 32.9°, 36.4 °, 38.9°, 44.8°, 50.6°, and 60.7° can be indexed to the (112), (200), (103), (211), (004), (220), (105), and (224) planes of the tetragonal spinel CoMn_2_O_4_ (JCPDS No. 77-0471), respectively. The peaks at 43.7° and 59.1° can be assigned to the (400) and (511) planes of the cubic MnCo_2_O_4_ (JCPDS No. 23-1237), respectively. The XRD pattern of Mn-Co spinel (Mn_1.5_Co_1.5_O_4_) deposited onto Ni metal foam reveals the formation of a dual-phase mixture of Mn_2_CoO_4_ and MnCo_2_O_4_ after calcination at 400 °C in air. The sharp peaks at 29.4°, 32.9°, 36.4 °, and 43.7° of (MCO/Ni)-15 min indicate that the tetragonal spinel Mn_1.5_Co_1.5_O_4_ is well crystallized. Based on a thermodynamic study of Mn_1+*x*_Co_2-*x*_O_4_ spinel, a single phase cubic spinel is stable for *x* < 0.3, cubic and tetragonal phases co-exist for 0.3 < *x* < 0.9, and a single phase tetragonal spinel is stable for *x* > 0.9, which is consistent with the observations in the present study [20].

#### 3.1.2. SEM Studies of MCO/Ni Electrodes

**Figure 2:**
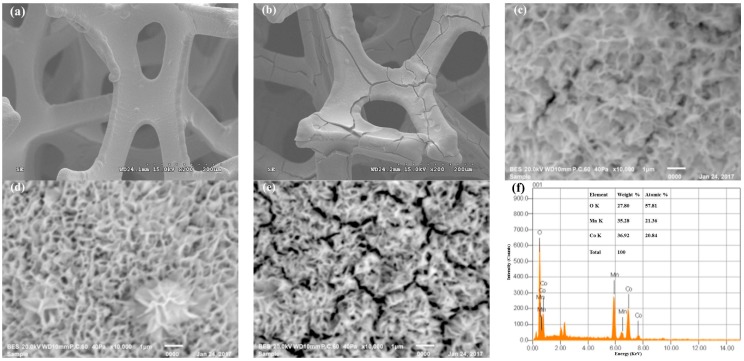
SEM images of (**a**) Ni MF (200×); (**b**) (MCO/Ni)-15 min (200×); (**c**) MCO deposited for 7.5 min; (**d**) MCO deposited for 15 min; and (**e**) MCO deposited for 30 min; and (**f**) EDS spectrum of MCO deposited for 15 min.

SEM images of bare Ni MF, the MCO/Ni composite electrode, and MCO deposited for various deposition times are shown in Figure 2. Figure 2a shows that Ni MF has a 3D macro-porous structure with a pore size of about 50–150 mm and interconnecting hole cells, leading to high porosity and a high specific surface area. After manganese-cobalt oxides were electrodeposited, covered arch ribs and a surface with cracks were observed for the (MCO/Ni)-15 min composite electrode (Figure 2b). Figure 2c–e show SEM images of MCO deposited for various deposition times on Ni MF. Figure 2c shows a SEM image of MCO deposited for 15 min, which consists of thin microflakes that are randomly interlaced, forming a nest-like porous structure. The morphology of MCO deposited for 7.5 min has a highly porous and loose structure and a lot of cracks formed for MCO deposited for 30 min (Figure 2d,e). As shown in Figure 2c–e, all electrodeposited MCO films have interconnected pores, which provide plenty of space for the transport of the electrolyte into the electrode material, thus increasing the liquid-solid interfacial area and the opportunity for Li^+^ insertion and extraction. Such a structure effectively utilizes electro-active materials and achieves excellent electrochemical performance. The energy dispersive spectroscopy spectrum (Figure 2f) shows the presence of Co, Mn, and O with an atomic ratio of Mn, Co, and O of ~1:0.98:2.71, further confirming the formation of the Mn_1.5_Co_1.5_O_4_ phase.

#### 3.1.3. Porosity and Surface Area Studies of MCO/Ni Electrodes

**Figure 3:**
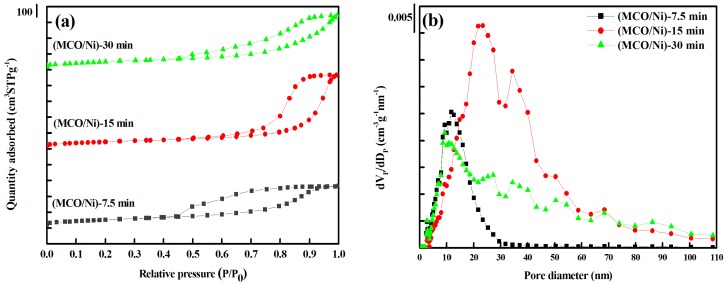
(**a**) N_2_ adsorption/desorption isotherms and (**b**) corresponding pore size distributions of (MCO/Ni)-7.5 min, (MCO/Ni)-15 min, and (MCO/Ni)-30 min.

The N_2_ adsorption-desorption isotherms of the MCO powders are shown in Figure 3a. All electrodeposited samples present a type IV isotherm with hysteresis loops, which corresponds to materials with a typical mesoporous structure. Compared to (MCO/Ni)-7.5 min, the inflection point in the isotherms of (MCO/Ni)-15 min shifted to a higher P/P_0_ range of 0.65–0.90, indicating an enlargement of pore size. A triangular shape and a steep desorption branch can be observed, which belong to an H_2_-type hysteresis loop, indicating the presence of mesopores with narrow mouths and wider bodies (ink-bottle pores) [21]. Figure 3b shows the corresponding pore size distributions of the obtained MCO powders. The (MCO/Ni)-7.5 min sample has a narrow pore size distribution centered at about 13 nm with a small portion extending to the mesopore scale. When the eletrodeposition time was increased to 15 or 30 min, the pore size distributions of the samples showed multimodal structures. A wide distribution of sizes over the mesopore/macropore boundary and extending to the macropore scale were observed for eletrodeposition times of 15 and 30 min. An eletrodeposition time of 15 min led to the largest and broadest pore size, as predicted from the obtained type IV isotherm. The sample eletrodeposited for 15 min had a wide pore size distribution, ranging from 3 to 110 nm, with bimodal pore size distributions centered at 25 and 37 nm, respectively, indicating a hierarchical porous structure. The textural characteristics of MCO samples and the Ni foam support are summarized in Table 1. Compared to the surface area, pore volume, and pore size of Ni foam, those of all composite samples were dramatically higher. The BET specific surface areas of (MCO/Ni)-7.5 min, (MCO/Ni)-15 min, and (MCO/Ni)-30 min were 185, 166, and 179 m^2^·g^−1^, respectively. The pore volume of (MCO/Ni)-15 min was larger than those of (MCO/Ni)-7.5 min and (MCO/Ni)-30 min. The average pore diameter values of samples deposited for 7.5, 15, and 30 min were 11, 16, and 8 nm, respectively. The large specific surface area and mesoporous/macroporous structure of Mn_1.5_Co_1.5_O_4_ on Ni foam contribute to fast ion (Li^+^) transport and reversible redox reactions, as discussed in the next section.

### 3.2. Electrochemical Studies of MCO/Ni Electrodes and PAC/(MCO/Ni) Asymmetric Supercapacitor Devices

**Figure 4:**
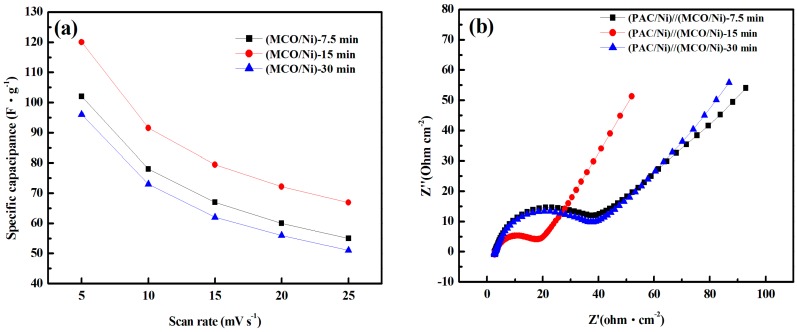
Cyclic voltammograms of (**a**) Plots of specific capacitance vs. potential scan rate of (MCO/Ni)-7.5 min, (MCO/Ni)-15 min, and (MCO/Ni)-30 min electrodes and (**b**) Nyquist plots of (PAC/Ni)//(MCO/Ni)-7.5 min, (PAC/Ni)//(MCO/Ni)-15 min, and (PAC/Ni)//(MCO/Ni)-30 min devices.

CV measurements of MCO/Ni electrodes were made individually prior to asymmetric supercapacitor fabrication, thus enabling the optimization of textural properties. The CV studies of the MCO/Ni electrodes were carried out in 9 M LiNO_3_ electrolyte with a working voltage of 0 to +1.0 V vs. SCE at five scan rates (5, 10, 15, 20, and 25 mV·s^−^^1^) using a three-electrode cell setup. Figure 4a shows the specific capacitances vs. voltage scan rate of the MCO/Ni electrodes. The (MCO/Ni)-15 min electrode exhibits the highest capacitance among the three electrodes for all scan rates. At a scan rate of 5 mV·s^−1^, the capacitance values of (MCO/Ni)-7.5 min, (MCO/Ni)-15 min, and (MCO/Ni)-30 min electrodes were 102, 120, and 96 F·g^−1^, respectively. The capacitance of (MCO/Ni)-30 min decreased to 51 F·g^−1^ at 25 mV·s^−1^; in contrast, (MCO/Ni)-15 min retained a capacitance of 67 F·g^−1^, nearly 1.3 times higher than that of (MCO/Ni)-30 min. The superior capacitive performance of (MCO/Ni)-15 min is due to its unique hierarchical porous structure, as shown by the BET results. A higher electrode/electrolyte contact area and a shorter diffusion length of Li^+^ ions can be obtained with a hierarchical porous structure, increasing specific capacity.

Moreover, EIS was used to investigate the combined resistance of the electrodes, electrolyte, and current collectors of the (PAC/Ni)//(MCO/Ni) asymmetric supercapacitor devices. It is well known that a high-frequency semicircle in a Nyquist plots is related to the charge transfer resistance and that a low-frequency line (the Warburg impedance) corresponds to capacitance behavior. As shown in Figure 4b, all Nyquist curves have a semicircle in the high-frequency region and a straight line in the low-frequency region. The intercept of the semicircle with the real axis is the equivalent series resistance, including the resistance of the electrolyte solution, the intrinsic resistance of the active material, and the contact resistance of the interface active material and current collector [22]. As can be seen, the equivalent series resistance values of the three devices are very similar (2.60, 2.72, and 2.70 Ω·cm^−2^, respectively). The diameter of the semicircle in the high-frequency region is directly related to the faradaic charge transfer resistance at the soild electrode/liquid electrolyte interface [23]. It can be seen that the (PAC/Ni)//(MCO/Ni)-15 min device has the smallest semicircle at higher frequency among the three samples (the estimated resistance values are 15.36, 34.35, and 34.40 Ω, respectively), indicating that it has the lowest faradaic charge transfer resistance, which is due to its hierarchical porous structure. The straight line in the low-frequency region is the Warburg impedance (Z_w_) and reflects the diffusive resistances resulting from the diffusion of redox species in the electrolyte, including electrolyte diffusion and proton diffusion [24]. The greater the angle of the straight line, the closer the capacitor behaves as an ideal supercapacitor [25]. (PAC/Ni)//(MCO/Ni)-15 min has the highest angle (above 45°), indicating more facile Li^+^ ion diffusion to the surfaces of the (MCO/Ni)-15 min electrode and the PAC electrode. Both the charge-transfer resistance and diffusive resistance of (PAC/Ni)//(MCO/Ni)-15 min are lower than those of (PAC/Ni)//(MCO/Ni)-7.5 min and (PAC/Ni)//(MCO/Ni)-30 min devices, which is attributable to the effective porous structure of the (MCO/Ni)-15 min electrode facilitating electron and ion transport.

**Figure 5:**
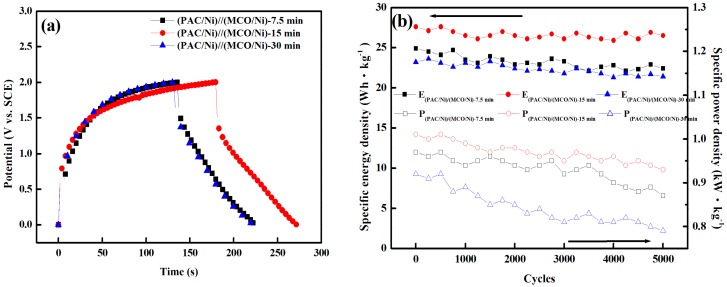
Performance of asymmetric PAC/Mn_1.5_Co_1.5_O_4_ device using MCO/Ni as positive electrode and PAC as negative electrode. (**a**) Charge-discharge curves of (PAC/Ni)//(MCO/Ni)-7.5 min, (PAC/Ni)//(MCO/Ni)-15 min, and (PAC/Ni)//(MCO/Ni)-30 min; and (**b**) Cycling stability test of (PAC/Ni)//(MCO/Ni)-7.5 min, (PAC/Ni)//(MCO/Ni)-15 min, and (PAC/Ni)//(MCO/Ni)-30 min devices.

GCD tests were performed for the fabricated (PAC/Ni)//(MCO/Ni)-7.5 min, (PAC/Ni)//(MCO/Ni)-15 min, and (PAC/Ni)//(MCO/Ni)-30 min devices. The discharge profiles of (PAC/Ni)//(MCO/Ni) devices consist of three regions: a rapid drop of voltage due to the internal resistance of MCO, a linear deviation of the time dependence of the voltage due to the EDLC of PAC, and slope variation of the time dependence due to the pseudo-capacitance behavior of MCO. The IR drop behaviors of (PAC/Ni)//(MCO/Ni)-7.5 min and (PAC/Ni)//(MCO/Ni)-30 min devices are similar, resulting from the similarity of their Warburg impedances (Z_w_) in the Nyquist plots. As shown in Figure 5a, there is an obvious flexion in the first cycle of the charge branch of each sample, indicating a significant contribution from pseudo-capacitance. The specific capacitances of (PAC/Ni)//(MCO/Ni) devices were calculated from the discharge branch of the GCD curves. The specific capacitance of (PAC/Ni)//(MCO/Ni)-15 min is 49.7 F·g^−1^, higher than those of (PAC/Ni)//(MCO/Ni)-7.5 min (44.9 F·g^−1^) and (PAC/Ni)//(MCO/Ni)-30 min (41.7 F·g^−1^). Moreover, the time required for charging and discharging was highest for (PAC/Ni)//(MCO/Ni)-15 min, which has higher specific energy and power density than those of (PAC/Ni)//(MCO/Ni)-7.5 min and (PAC/Ni)//(MCO/Ni)-30 min.

Long-term cycling GCD tests, an important criterion of supercapacitors for energy storage applications, were performed to further investigate the stability performance of the obtained devices. The GCD experiments were carried out in the voltage range of 0 to 2.0 V with a constant current density of 1 A·g^−1^ over 5000 cycles. As shown in Figure 5b, the (PAC/Ni)//(MCO/Ni)-15 min device had the lowest degradation of specific energy density and specific power density. A slight loss (≈4%) of the initial specific energy density and a slight loss (≈8%) of the initial specific power density were observed after 5000 cycles for the (PAC/Ni)//(MCO/Ni)-15 min device, demonstrating excellent long-term electrochemical stability. The specific energy density and specific power density of (PAC/Ni)//(MCO/Ni) asymmetric supercapacitors in the 1st and 5000th cycle are listed in Table 2. Although the (PAC/Ni)//(MCO/Ni)-7.5 min device was stable (~10% loss in energy density and ~10% loss in power density) after 5000 cycles, its low energy density is a drawback. For the (PAC/Ni)//(MCO/Ni)-30 min device, a ~14% drop in power density after 5000 cycles was observed. The electrodeposition time used for the MCO/Ni electrode, thus, greatly affects the performance of an asymmetric supercapacitor device. For (PAC/Ni)//(MCO/Ni)-30 min, the fully coated Mn_1.5_Co_1.5_O_4_ deposited onto Ni metal foam increased the internal resistance of the electrode. Two things contributed to the excellent long-term electrochemical stability of the (PAC/Ni)//(MCO/Ni)-15 min device. The first is the lower charge-transfer resistance and diffusive resistance of (PAC/Ni)//(MCO/Ni)-15 min compared to those of (PAC/Ni)//(MCO/Ni)-7.5 min and (PAC/Ni)//(MCO/Ni)-30 min electrodes. The second is the high utilization of the active material and excellent reversibility in faraday reactions resulting from the relatively high surface area and hierarchical mesoporous structure of the (MCO/Ni)-15 min electrode. The hierarchical pores with a large pore volume of the (MCO/Ni)-15 min electrode not only serve as a reservoir for the electrolyte, but also enhance the ion transport and proton diffusion kinetics in the interior of the electrode. Hao reported that a hierarchical porous structure of the electrode material could be beneficial for high-efficiency supercapacitors due to macropores acting as an ion-buffering reservoir, mesopores facilitating ion transport, and micropores facilitating charge storage [26]. In particular, a large pore volume can effectively mitigate the volume change that occurs during the repeated insertion and extraction of Li^+^ ions, which can stabilize the integrity of the positive electrode, thus improving device cyclability [27,28].

Table 3 shows a comparison of the performance of the PAC/MCO-Ni supercapacitor fabricated in the present study with supercapacitors from the literature. Shivakumara et al. [29] used reduced graphene oxide (RGO) to fabricate symmetric RGO/RGO supercapacitors in a non-aqueous electrolyte. The RGO/RGO supercapacitors exhibited a very high specific energy of 44 Wh·kg^−1^ at 0.15 kW·kg^−1^. Lin et al. [7] used MnFe_2_O_4_ powders and LiMn_2_O_4_ powders as the active materials for the negative and positive electrodes, respectively. The asymmetric supercapacitors had specific cell energies of 10 and 5.5 Wh·kg^−1^ at 0.30 and 1.80 kW·kg^−1^, respectively. Li et al. [30] fabricated α-MnOx/GS-CNT composite electrodes by depositing α-MnO_x_ on a graphene sheet-carbon nanotube (GS-CNT) substrate. They exhibited an excellent energy of 46 Wh·kg^−1^ at 33.20 kW·kg^−1^. Kim et al. [31] developed AC/SiC-N-MnO_2_ asymmetric supercapacitors with AC and a silicon carbide-MnO_2_ nanoneedle (SiC-N-MnO_2_) as the negative and positive electrodes, respectively. The asymmetric supercapacitors exhibited a high energy density of 30 Wh·kg^−1^ at 0.11 kW·kg^−1^. Tsai et al. [32] prepared AC/MnO_2_-Ni asymmetric supercapacitors with AC and MnO_2_ deposited on a Ni metal foam substrate (MnO_2_-Ni) as the negative and positive electrodes, respectively. The asymmetric supercapacitors had an energy density of 9 Wh·kg^−1^ at 0.71 kW·kg^−1^. Based on the literature survey, high-energy-density supercapacitors can, thus, be achieved using graphene, CNTs, and RGO [29,30]. Although various carbon-based and metal oxide materials have been utilized for asymmetric supercapacitors, the energy and power densities of supercapacitors still need to be improved. In the present study, low-cost materials, including commercial AC and mesoporous Mn_1.5_Co_1.5_O_4_ spinel deposited on Ni metal foam, were used to fabricate asymmetric supercapacitors. The asymmetric supercapacitors exhibited a high energy density of 28 Wh·kg^−1^ at 1.01 kW·kg^−1^, for a power density that is higher than some previously reported values [7,29,31].

## 4. Conclusions

Mesoporous Mn_1.5_Co_1.5_O_4_/Ni foam composite electrodes were fabricated via the electrodeposition method. BET results indicate that the deposition time significantly affects the textural properties of MCO films and the electrochemical properties of (PAC/Ni)//(MCO/Ni) devices. The hierarchical structure of the (MCO/Ni)-15 min electrode not only reduces faradaic charge transfer resistance and diffusive resistance, but also mitigates volume change during charge/discharge cycling. Due to the hierarchical porous structure of the electrode and the use of a suitable electrolyte, the (PAC/Ni)//(MCO/Ni)-15 min device had a specific power density of 1.01 kW·kg^−1^ and a specific energy of 27.6 Wh·kg^−1^. After 5000 cycles, the specific energy density retention and power density retention were 96% and 92%, respectively, suggesting exceptional cycling stability. The LiNO_3_ electrolyte was entrapped in the CMC polymer matrix, enhancing cycling stability and allowing a sufficient voltage window. The combination of an active electrode material with a suitable electrolyte may facilitate the development of energy storage systems with high performance and high energy density.

## Figures and Tables

**Table 1:** Textural properties of Ni MF, (MCO/Ni)-7.5 min, (MCO/Ni)-15 min, and (MCO/Ni)-30 min.

Sample	*S*_BET_ (m^2^·g^−1^)	*V*_pore_ (cm^3^·g^−1^)	*D*_p_ (nm)
Ni MF	2	0.0003	2
(MCO/Ni)-7.5 min	185	0.53	11
(MCO/Ni)-15 min	166	0.73	16
(MCO/Ni)-30 min	179	0.40	8

*S*_BET_: specific surface area; *V*_pore_: pore volume; *D*_p_: pore diameter.

**Table 2:** Specific energy density and specific power density of (PAC/Ni)//(MCO/Ni**)** asymmetric supercapacitors in 1st and 5000th cycles.

Asymmetric Supercapacitor	E_1st_^a^ (Wh·kg^−1^)	P_1st_^b^ (kW·kg^−1^)	E_5000cycle_^c^ (Wh·kg^−1^)	P_5000cycle_^d^ (kW·kg^−1^)
(PAC/Ni)//(MCO/Ni)-7.5 min	24.9	0.97	22.4	0.87
(PAC/Ni)//(MCO/Ni)-15 min	27.6	1.01	26.5	0.93
(PAC/Ni)//(MCO/Ni)-30 min	23.2	0.92	21.4	0.79

^a^ E_1st_, specific energy density for 1st cycle calculated from discharge branch of GCD curves at constant current density of 1 A·g^−1^; ^b^ P_1st_, specific power density for 1st cycle calculated from discharge branch of GCD curves at constant current density of 1 A·g^−1^; ^c^ E_5000cycle_, specific energy density at 5000th cycle calculated from discharge branch of GCD curves at constant current density of 1 A·g^−1^; ^d^ P_5000cycle_, specific power density at 5000th cycle calculated from discharge branch of GCD curves at constant current density of 1 A·g^−1^.

**Table 3:** Comparison of carbon-based and metal oxide electrodes used for supercapacitors.

Materials	Energy Density (Wh·kg^−1^)	Power Density (kW·kg^−1^)	Reference
RGO/RGO	44	0.15	[29]
MnFe_2_O_4_/LiMn_2_O_4_	10	0.30	[7]
α-MnO*_x_*/GS-CNT	46	33.20	[30]
AC/SiC-N-MnO_2_	30	0.11	[31]
AC/MnO_2_-Ni	9	0.71	[32]
PAC/Mn_1.5_Co_1.5_O_4_-Ni	28	1.01	This work
